# Non-uniformity smoothing of direct-driven fuel target implosion by phase control in heavy ion inertial fusion

**DOI:** 10.1038/s41598-019-43221-7

**Published:** 2019-04-30

**Authors:** R. Sato, S. Kawata, T. Karino, K. Uchibori, A. I. Ogoyski

**Affiliations:** 1 Graduate School of Engineering, Utsunomiya University, Utsunomiya, Japan; 2 Department of Physics, Varna Technical University, Varna, Bulgaria

**Keywords:** Computational science, Plasma physics, Energy infrastructure

## Abstract

We have proposed a dynamic smoothing method based on a phase control to smooth plasma non-uniformities in perturbed plasma systems. In this paper, the dynamic smoothing method is applied to a spherical direct-driven fuel target implosion in heavy ion inertial confinement fusion. We found that the wobbling motion of each heavy ion beam (HIB) axis induces a phase-controlled HIBs energy deposition, and consequently the phase-controlled implosion acceleration is realized, so that the HIBs irradiation non-uniformity is successfully smoothed. HIB accelerators provide a well-established performance to oscillate a HIB axis at a high frequency. In inertial confinement fusion, a fuel implosion uniformity is essentially significant for achieving the DT fuel compression and for releasing the fusion energy, and the non-uniformity of the implosion acceleration should be less than a few %. The results in this paper demonstrate that the wobbling HIBs would provide an improvement in the fuel target implosion uniformity.

## Introduction

In inertial confinement fusion, the fusion fuel should be compressed to a high density to reduce an input driving energy^1,2^. In order to realize an inertial confinement fusion (ICF) system, a sufficient fusion energy output is required, and a uniform fuel implosion is essentially required to release the fusion output energy. For the uniform implosion of the fusion fuel, the non-uniformity of the implosion acceleration should be less than a few %^3,4^. On the other hand, a dynamic smoothing of plasma non-uniformities was proposed in refs^5–8^. In this paper the dynamic smoothing method is applied to a spherical DT fuel target implosion in heavy ion inertial confinement fusion (HIF), and two-dimensional fluid simulations are performed to investigate the dynamic smoothing effect on the implosion non-uniformity of the DT fuel spherical pellet, illuminated by heavy ion beams (HIBs). In our study each HIB axis oscillates at a high frequency, for example, a few hundred MHz or higher in order to realize the dynamic smoothing^5–9^. We found that the wobbling motion of each HIB axis induces a phase-controlled HIBs energy deposition, and consequently the phase-controlled implosion acceleration is realized, so that the HIBs illumination non-uniformity is successfully mitigated. The HIB accelerators provide a well-established performance to oscillate a HIB axis at a high frequency. In refs^5,6,8^ we have proposed the dynamic smoothing mechanism and applied it to reduce the Rayleigh-Taylor instability (RTI) growth by using the beam axis wobbling. References 8 and 9 also showed that the dynamic smoothing mechanism would be rather robust against the perturbation in the driver dynamic behavior. The smoothing mechanism was also applied to the filamentation instability in ref.^7^. The results in refs^5–8^ presented the basic idea and its applications to the RTI and filamentation instabilities. In ref.^9^ we also applied the dynamic smoothing mechanism to smooth the HIBs deposition-energy non-uniformity in a HIF fuel pellet material, and found that the wobbling HIBs help mitigate the HIBs-energy deposition non-uniformity in the HIBs-energy absorber layer of a HIF fuel target. However, in the previous works in refs^5–9^ we could not yet reach to present the dynamic smoothing by the oscillating behavior of the fuel implosion acceleration. In this paper we found that the HIBs wobbling behavior controls the implosion acceleration oscillation and also contributes to reduce the implosion non-uniformity.

Here we briefly present the dynamic smoothing mechanism in perturbed plasmas^5–9^. In order to control physical systems, like tall buildings, the feedback control is well known and employed widely to stabilize the systems. In the feedback control the perturbation amplitude and phase are measured, and another perturbation with the reverse phase is applied actively to compensate the original perturbation. In plasmas we cannot measure the amplitude and the phase of the plasma perturbation. Therefore, usually the instability growth rate is discussed in plasma science. However, if the perturbation phase is actively imposed by a driving source wobbling or oscillation, the amplitude of the perturbation can be controlled in the same way^5–10^ as the usual control theory. For example, the driving particle-beam axis wobbling mitigates the growth^7^ of the filamentation instability^11–14^. The oscillating beam induces the phase-defined continuous perturbations. The growth of the integrated instability amplitude is mitigated. In HIF the HIB axis can be also wobbled in the heavy ion accelerator with a high frequency^15–18^. In refs^15,18^, a couple of biased electrodes are employed to produce the HIB wobbling behavior at a high frequency. The amplitude and phase of the perturbation applied are defined by the HIB axis oscillation behavior. The dynamic smoothing mechanism was also applied to smooth the HIBs deposition energy non-uniformity^5,6,8,9^.

In this research, we propose to employ the wobbling HIBs to reduce the HIB implosion nonuniformity. First, we shortly summarize the dynamic smoothing mechanism in plasmas, and in Sec. III it is applied to the HIF fuel target implosion. In this paper we do not study the RTI in HIF but focus on an overall implosion uniformity in HIF. The results demonstrate that the dynamic smoothing mechanism would improve the fuel target implosion uniformity by the wobbling HIBs in HIF.

## Dynamic Smoothing Mechanism

**Figure 1:**
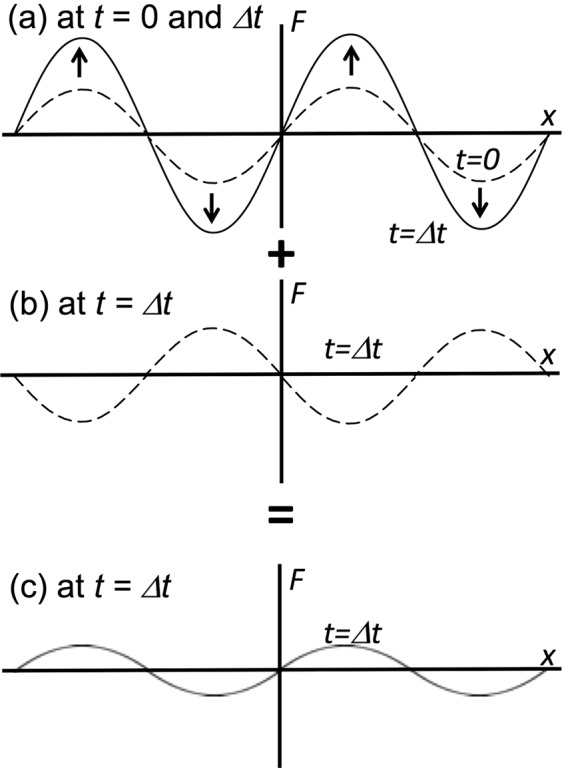
Concept of the dynamic smoothing mechanism. (**a**) At *t* = 0 a perturbation is imposed. The initial perturbation grows with *γ* for an unstable system. (**b**) After *Δt* another perturbation, which has an inverse phase, is actively imposed. (**c**) After the superimposition of the perturbations in (**a**,**b**) at *Δt*, the actual perturbation amplitude is mitigated well.

First, we consider a perturbed plasma system, which has a single mode of $k=2\pi /\lambda$ with the amplitude of $a={a}_{0}{e}^{ikx+\gamma t}$. For a stable system *γ* is negative, and for an unstable system *γ* means the instability growth rate. Here *λ* is the wave length. Figure 1(a) shows an example initial perturbation in an unstable system. The initial perturbation in the unstable system is assumed to be imposed at *t* = 0, and the perturbation grows with the growth rate of *γ*. If the next perturbation is actively superimposed on the system at *t* = *Δt*, and also if the perturbation added has the inverse phase as shown in Fig. 1(b), the integrated amplitude growth is mitigated (see Fig. 1(c)). An ideal dynamic smoothing mechanism is demonstrated in Fig. 1 ^5–7^.

It is difficult to detect the perturbation phase and amplitude in plasmas. However, as presented in Fig. 1, if the energy driver, which may have perturbations, provides a wobbling motion, we can expect a control of the perturbation amplitude growth. For example, in a heavy ion accelerator the ion beam axis can be oscillated in a controlled manner to realize the wobbling or oscillating behavior^15,16,18^.

A superimposed perturbation for a physical quantity *F* at *t* = *τ* may be expressed as follows:

1 $$F=\delta F{e}^{i{\rm{\Omega }}\tau }{e}^{\gamma (t-\tau )+i\overrightarrow{k}\cdot \overrightarrow{x}}.$$

Here we assume the uniform oscillation of the perturbed driver in time. In Eq. (1) the amplitude is described by *δF*, *Ω* shows the wobbling frequency of the driving beam, and *Ωτ* is the phase shift of the perturbations superimposed. The integrated actual perturbation at *t* is derived as follows:

2 $${\int }_{0}^{t}d\tau \,\delta F{e}^{i{\rm{\Omega }}\tau }{e}^{\gamma (t-\tau )+i\overrightarrow{k}\cdot \overrightarrow{x}}\propto \,\frac{|\gamma |+i{\rm{\Omega }}}{{\gamma }^{2}+{{\rm{\Omega }}}^{2}}\delta F{e}^{\gamma t}{e}^{i\overrightarrow{k}\cdot \overrightarrow{x}}$$

When γ ≤ 0, the system is stable and Eq. (2) shows a simple dynamic smoothing of the perturbations. When γ ≥ 0 and ${\rm{\Omega }}\gg \gamma$ for the unstable system, the amplitude reduction ratio is γ/Ω^5,6,8^. Even for ${\rm{\Omega }}\cong \gamma$ we can still expect the significant mitigation. At this point, it should be noted that the integrated perturbation amplitude is mitigated well, but the growth rate *γ* of the instability does not change. The result in Eq. (2) suggests that the wobbling frequency *Ω* should be high compared with the instability growth rate of *γ* for the effective mitigation of the integrated perturbation amplitude.

## Fuel Target Implosion Driven By Wobbling HIBs

**Figure 2:**
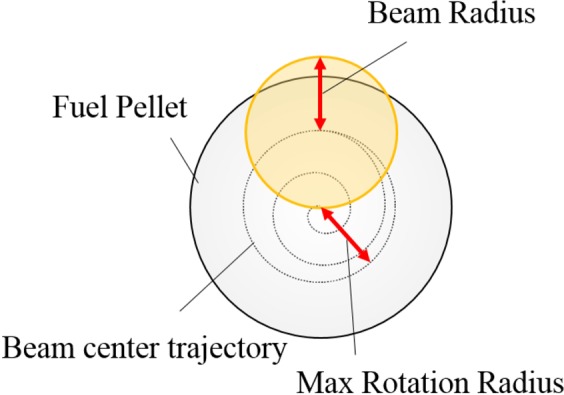
Schematic diagram for the spiral wobbling heavy ion beam. The beam center starts from the center, and moves spirally until *t* = 2.0τ_wb_ at which the rotation radius becomes 0.9 mm in this paper.

The wobbling HIBs would introduce a little successive oscillating perturbation onto an inertial fusion fuel target implosion. Therefore, the HIBs irradiation non-uniformity can be mitigated by the superimposition of phase-controlled perturbations in heavy ion inertial fusion (HIF)^2,5,6^. The oscillating non-uniform acceleration field is obtained by the HIBs’ axes oscillation. At the same time, even in the stable phase of the fuel implosion the wobbling behavior smooths the HIBs illumination non-uniformity, as shown in Sec. II. The wobbling HIB as an irradiation driver beam onto a DT fuel pellet is shown in Fig. 2 schematically with the spiral wobbling beam trajectory^17^, which reduces the initial imprint of the HIB-induced non-uniformity. For the spiral wobbling beam employed in this paper, the beam radius in the fuel pellet surface changes from 3.8 mm to 3.7 mm at 1.3 *τ*_wb_. Here *τ*_wb_ is one rotation time interval of the beam axis oscillation. The last HIB rotation radius becomes 0.9 mm at *t* = 2.0 *τ*_wb_. When we employ the spiral motion of each HIB axis, the initial imprint of the HIB irradiation non-uniformity is significantly reduced^17^.

**Figure 3:**
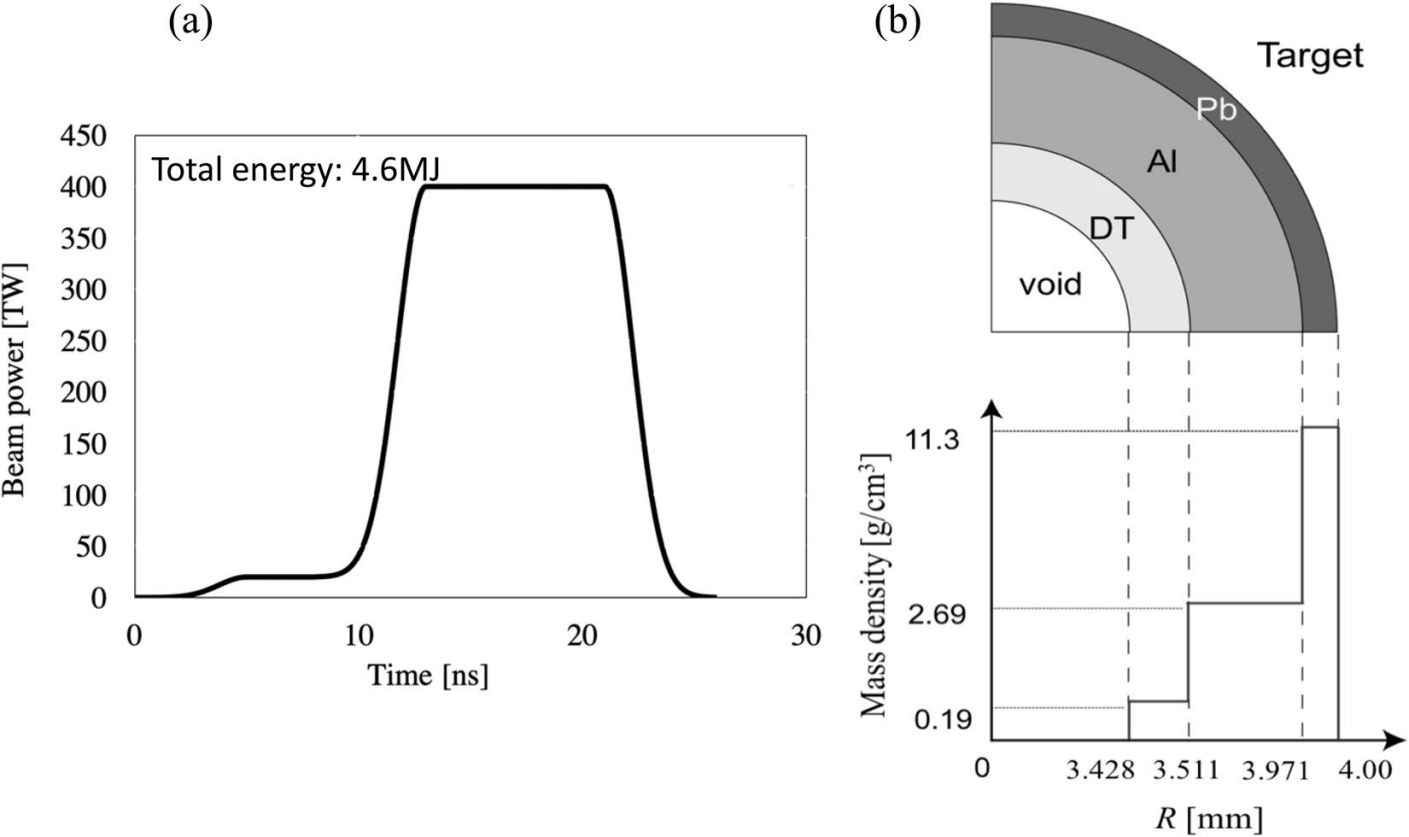
(**a**) Input HIB pulse. The HIB pulse consists of the foot pulse and the main pulse. (**b**) DT fuel pellet structure employed in this paper. The DT total mass is 2.4 mg in this paper.

Figure 3 show (a) the HIBs input pulse designed and (b) the fuel target structure used in this work. The HIB input pulse consists of the low-power foot part and the higher power main part: the peak power of the foot pulse is 22TW with the initial rising time of 5 ns, and the main pulse rises at 8 ns and reaches 400TW at 13 ns with the 5 ns rising and decaying times. The HIBs irradiation is terminated at 25.9 ns, and the total input energy is 4.6MJ. The spiral trajectory in Fig. 2 is also introduced to the main pulse in order to reduce the initial imprint of the HIBs irradiation nonuniformity due to the pulse power rising while the beam is wobbling. The same HIB spiral trajectory in Fig. 2 starts again at 10.5 ns. In this work we employ Pb^+^ ions as HIB ions. The Pb^+^ beam particle energy is 8 GeV, and each HIB has the Gaussian profile in the cross section. The total HIB number is 32 in this work^19,20^, and the HIBs illumination scheme in ref.^20^ is employed for the 32 HIBs^19–23^. As shown in Fig. 2(b), the target outer radius is 4 mm, and the HIBs irradiation energy loss appears by the final beam radius 3.7 mm with a 0.9 mm rotation radius. Therefore, a small part of the HIB ions does not hit the target, and throughout the work in this paper the HIBs maximum energy loss is about 7.3%. In this paper two-dimensional implosion simulations based on the three-temperature fluid model^24–27^ are performed to investigate the dynamic smoothing effect on the implosion uniformity of the DT fuel spherical pellet, illuminated by the wobbling HIBs. The implosion fluid simulation code is coupled to a detail energy deposition code for the HIBs illumination^21–23^.

The details of the 2D computer simulation code is presented in ref.^27^: the 2D implosion code system is an integrated direct-driven DT fuel implosion code in HIF, and consists of four parts: the HIBs illumination code^21–23^, the Lagrange fluid code, the data conversion code from the Lagrange code to the Euler code, and Euler code. The fluid model is the three-temperature model in ref.^26^. In the Lagrange fluid code the spatial meshes move together with the fluid motion. However the mass and energy conservations are well described, the Lagrange meshes cannot follow the fluid large deformation. On the other hand, the Euler meshes are fixed to the space, and the fluid moves through the meshes. Therefore, just before the void closure time, that is, the stagnation phase, the Lagrange code is used to simulate the DT fuel implosion. After the void closure time, the Euler code is employed to simulate the DT fuel further compression, ignition and burning. Between the Lagrange code and the Euler code the data should be converted by the data conversion code. In our simulations we employ the *r*-*z* coordinate. In the Lagrange code, 82 meshes in the radial direction and 90 meshes in the theta direction are used. In the Euler code 136~286 meshes in the *r* direction and 272~560 meshes in the *z* direction are used. In the simulation results presented in this paper, the spatial resolution near the stagnation phase in the Euler code is about 4~7 μm/mesh, depending on the target parameters. By the HIBs illumination code^21–23^ we obtain the 3D HIBs energy deposition, from which the 2D sliced deposition energy profile is obtained. The sliced HIBs energy deposition profile is employed for the simulations in the Lagrange code. Therefore, the wobbling frequency and the wobbling HIBs motion are reflected in the fluid implosion code.

**Figure 4:**
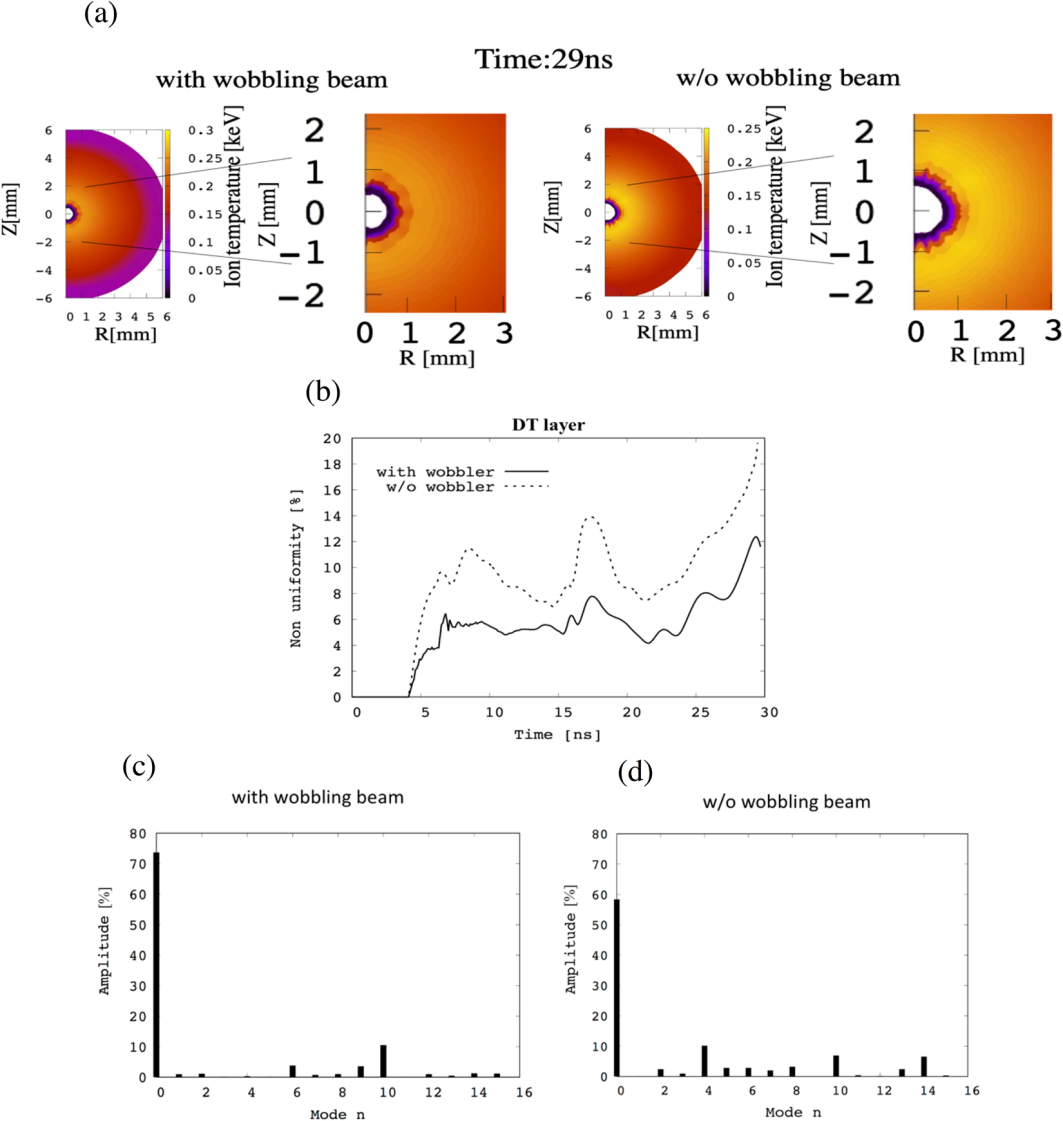
Implosion results of the fuel target ion temperature. (**a**) Ion temperature distributions at t = 29 ns before the void closure with the spiral wobbling beam (left) and without the wobbling beam (right). (**b**) The RMS nonuniformity histories of the DT ion temperature with the spiral wobbling beam (the solid line) and without the wobbler (the dotted line). The Legendre-function mode (*n*) analyses of the ion temperature non-uniformity in [%] of the DT layer are shown in (**c**) with and (**d**) without the wobbler at 25 ns.

First, the ion temperature distributions are shown at *t* = 29 ns in Fig. 4(a) for the wobbling HIBs with the rotation frequency of 424 MHz (the left figure of Fig. 4(a)) and without the wobbler (the right figure of Fig. 4(a)). The HIBs deposit their energy mainly in the Al layer through the Bragg Peak characteristic, and Fig. 4(a) confirm the mitigation of the implosion non-uniformity from the wobbling HIBs. Figure 4(b) shows the root-mean-square (RMS) non-uniformity histories of the target ion temperature. The non-uniformity is evaluated by the total relative RMS. Figure 4(b) also presents that the implosion non-uniformity of the DT fuel target is reduced well by the spiral wobbling HIBs. Figure 4(c,d) display the non-uniformity mode analysis results for the DT ion temperature at *t* = 25 ns based on the Legendre function^28^. The mode *n* = 0 pictures the perfect spherical shape of *P*_0_. All the results in Fig. 4 confirm that the wobbling motion of the HIBs axes contribute efficiently to the HIBs irradiation non-uniformity smoothing.

**Figure 5:**
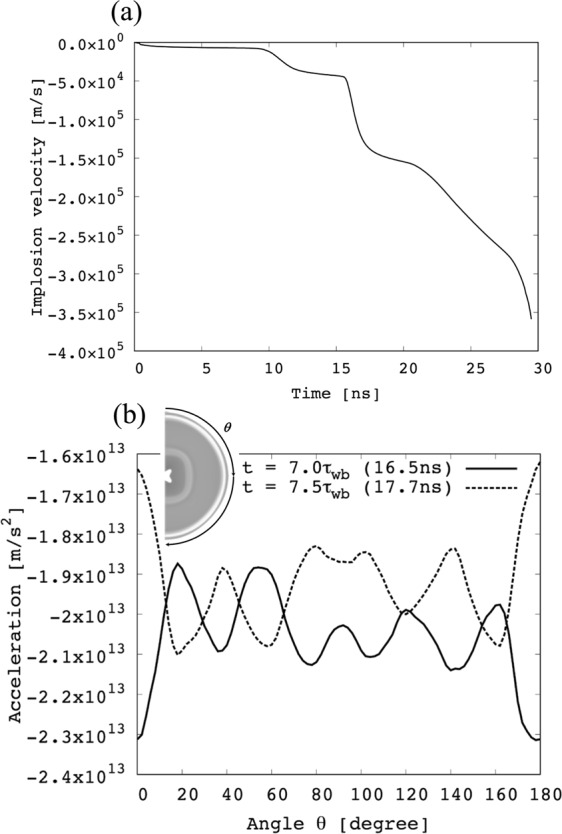
Shown here are (**a**) the spatially-averaged DT implosion speed, and (**b**) the spatial distributions of the DT implosion acceleration at *t* = 7.0 *τ*_wb_ and 7.5 *τ*_wb_ along the polar angle of *θ*. The DT implosion acceleration reflects the HIBs wobbling motion successfully. Consequently, it is expected that the dynamic smoothing mechanism works to reduce the HIBs illumination non-uniformity.

Figure 5(a) shows the averaged DT implosion speed, and Fig. 5(b) the DT implosion acceleration at *t* = 7.0 τ_wb_ and 7.5 *τ*_wb_ along the polar angle of *θ* of the target. In this specific case the wobbler rotation time is *τ*_wb_ = 2.36ns. Figure 5(b) demonstrates that the DT fuel implosion acceleration reflects adequately the HIBs wobbling behavior: the wobbling HIBs deposit their energy almost in the inner layer of the Al layer (see Fig. 3(b)), and the DT layer is accelerated inward with a high acceleration (see Fig. 5). The wobbling HIBs deposit their energy with a small non-nonuniformity, and the spatial profile of the implosion acceleration oscillates in time as shown in Fig. 5(b) as expected. The dynamic smoothing mechanism shown in Sec. II is realized successfully.

**Figure 6:**
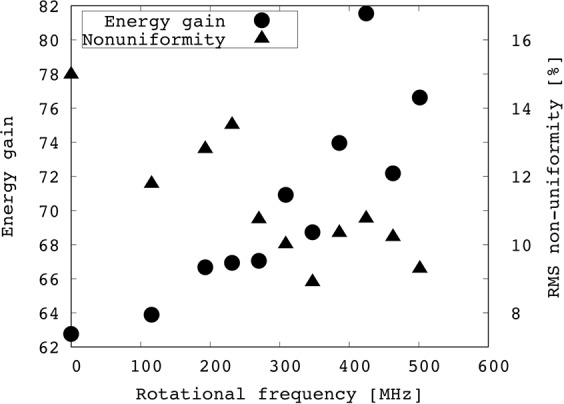
Energy gain and ion temperature RMS nonuniformity of DT layer at *t* = 28 ns versus the rotational frequency of the wobbling HIB axis.

Figure 6 shows the energy gain (solid circle) and the RMS non-uniformity (solid triangle) for the DT ion temperature versus the rotational frequency of the wobbling HIB axis. The rotational frequency changes from 0 to 500 MHz. Figure 6 features the remarkable improvement of the implosion uniformity with the high rotation frequency, when the wobbling HIB rotation frequency become higher than ~200 MHz. The RMS non-uniformity also shows an overall tendency of the smoothing effect for the wobbling HIBs on the HIBs illumination non-uniformity.

**Figure 7:**
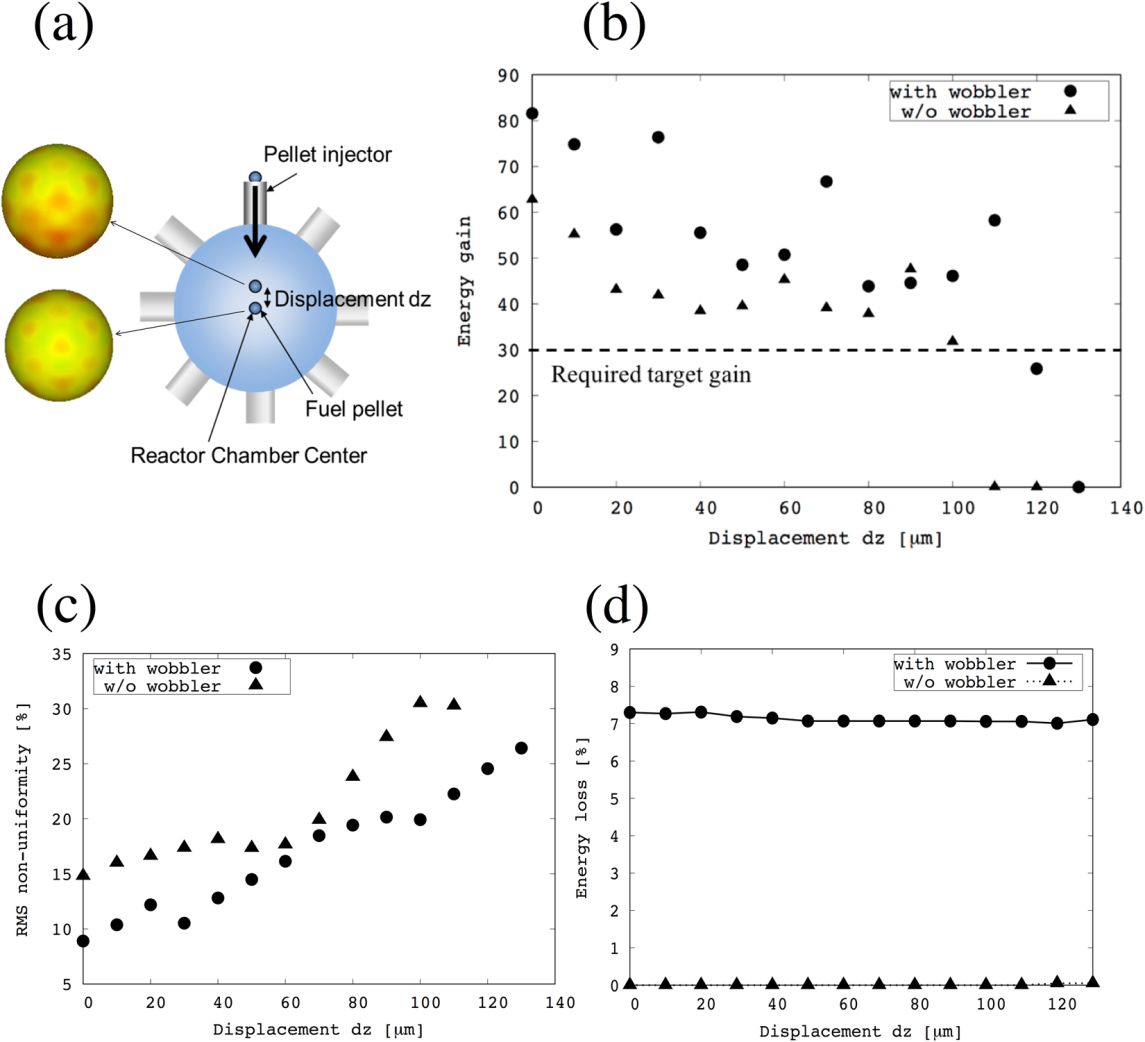
(**a**) Schematic diagram for the fuel-pellet alignment error *dz* in a fusion reactor chamber is presented. A fuel pellet is injected from the outside of the reactor to the reactor center. The HIBs illumination non-uniformity is enhanced by the alignment error *dz*. (**b**) Shows the fusion energy gain versus the target alignment error *dz*. The solid circles show the energy gain for the wobbling HIBs irradiation, and the triangles present the gain for the HIBs without the wobbling motion. In (**c**), the RMS non-uniformities for the DT ion temperature versus *dz* are shown at t = 28 ns for the cases with the wobbling HIBs (circles) and without the wobbling behavior (triangles). (**d**) Shows the HIB mishitting energy loss versus *dz* with the wobbling motion (circles) and without the wobblers (triangles).

In an actual inertial fusion reactor a fuel target alignment error (*dz*) may appear, when a fuel target is injected from the outside of a fusion reactor and is aligned at the center of the fusion reactor (see Fig. 7(a)). The fuel target alignment error would induce and enhance the HIBs irradiation non-uniformity on the fuel target, and the HIBs irradiation non-uniformity leads to a degradation of the fusion energy output. Figure 7(b) displays that the fusion energy gain versus the fuel target alignment error *dz* with and without the HIBs wobbling behavior. The DT fuel ignition is not achieved beyond about *dz* = 100 μm for the non-wobbling HIBs. However, the fuel target is ignited up to *dz* ~120 μm by the irradiation of the wobbling HIBs as shown in Fig. 7(b). The required target gain for the fusion power reactor is >~30 in HIF due to the HIB driver efficiency of 30~40%^2^. Therefore, the target alignment error *dz* of about 110μm would be tolerable to release the sufficient fusion energy in HIF. Figure 7(c) shows the RMS non-uniformity at *t* = 28 ns for the DT ion temperature versus the target misalignment *dz* for the cases with the wobbling HIBs (circles) and without the wobbling HIBs (triangles). The non-uniformity increases with the increase in *dz*, and the HIBs wobbling behavior contributes to mitigate the HIBs illumination non-uniformity. In Fig. 7(d), the HIB ion mishitting ratio is presented against the target alignment error *dz* for the wobbling HIBs (circles) and without the wobbling HIBs (triangles). As shown in Fig. 2, the target outer radius is 4 mm in this paper, and the HIBs irradiation energy loss appears for the final beam radius of 3.7 mm with a rotation radius of 0.9 mm. Therefore, a small part of the HIB ions does not hit the target, and the HIBs maximum energy mishitting loss is about 7.3%.

## Conclusions and Summary

We have presented the dynamic smoothing of the target implosion non-uniformity, originated from the HIBs illumination non-uniformity, through the spiral wobbling HIBs in HIF. The target implosion non-uniformity is mitigated successfully by the wobblers.

The dynamic smoothing mechanism employed in this paper and described in Sec. II is based on the phase control of the non-uniformities introduced. In plasmas it is difficult to realize the feedback control, because we cannot measure the perturbation phase and pertaining amplitude in plasmas. However, we can control the phases of the plasma perturbations which are actively imposed from the outside of the systems. For instance, the drivers, like the wobbling HIBs in HIF introduced in this paper or a wobbling electron beam in ref.^7^, would introduce their own non-uniformities. The phases of the non-uniformities imposed by the drivers are controlled, for example, by the wobbling motion, and consequently the dynamic smoothing mechanism can be implemented in plasmas. Therefore, the dynamic smoothing mechanism may be applied to various plasma instabilities and non-uniformities, as far as the phase of the non-uniformities are controllable. As shown in ref.^17^, a wobbling device is being planned to be installed in a HIB accelerator. In several year the dynamic smoothing mechanism may be studied experimentally.

We also have to point out that the dynamic smoothing mechanism is not almighty. If the non-uniformity phase cannot be controlled actively, the mechanism cannot be realized. For example, if the DT fusion pellet has an unexpected aspherical shape or if the fusion pellet has a non-uniformity in the shell thickness, the dynamic smoothing mechanism does not work. In addition, it would be better to point out again that the present results shown in this paper demonstrate that the wobbling HIBs help reduce the HIBs illumination non-uniformity in HIF. The growth rate *γ* of the plasma instability does not change as shown in Eq. (2). Therefore, the results in this paper come from the mitigation of the perturbation amplitude.
